# Dynamic nitrogen fixation in an aerobic endophyte of *Populus*

**DOI:** 10.1093/ismejo/wrad012

**Published:** 2024-01-10

**Authors:** Andrew W Sher, Jayde A Aufrecht, Daisy Herrera, Amy E Zimmerman, Young-Mo Kim, Nathalie Munoz, Jesse B Trejo, Vanessa L Paurus, John B Cliff, Dehong Hu, William B Chrisler, Robert J Tournay, Emma Gomez-Rivas, Galya Orr, Amir H Ahkami, Sharon L Doty

**Affiliations:** School of Environmental and Forest Sciences, College of the Environment, University of Washington, Seattle, WA, 98195-2100, United States; Environmental Molecular Sciences Laboratory, Pacific Northwest National Laboratory, Richland, WA, 99354, United States; Environmental Molecular Sciences Laboratory, Pacific Northwest National Laboratory, Richland, WA, 99354, United States; Biological Sciences Division, Pacific Northwest National Laboratory, Richland, WA, 99354, United States; Biological Sciences Division, Pacific Northwest National Laboratory, Richland, WA, 99354, United States; Environmental Molecular Sciences Laboratory, Pacific Northwest National Laboratory, Richland, WA, 99354, United States; Biological Sciences Division, Pacific Northwest National Laboratory, Richland, WA, 99354, United States; Biological Sciences Division, Pacific Northwest National Laboratory, Richland, WA, 99354, United States; Environmental Molecular Sciences Laboratory, Pacific Northwest National Laboratory, Richland, WA, 99354, United States; Environmental Molecular Sciences Laboratory, Pacific Northwest National Laboratory, Richland, WA, 99354, United States; Environmental Molecular Sciences Laboratory, Pacific Northwest National Laboratory, Richland, WA, 99354, United States; School of Environmental and Forest Sciences, College of the Environment, University of Washington, Seattle, WA, 98195-2100, United States; School of Environmental and Forest Sciences, College of the Environment, University of Washington, Seattle, WA, 98195-2100, United States; Environmental Molecular Sciences Laboratory, Pacific Northwest National Laboratory, Richland, WA, 99354, United States; Environmental Molecular Sciences Laboratory, Pacific Northwest National Laboratory, Richland, WA, 99354, United States; School of Environmental and Forest Sciences, College of the Environment, University of Washington, Seattle, WA, 98195-2100, United States

**Keywords:** endophyte, nitrogen fixation, Populus, plant–microbe interactions, 15 N-tracking proteomics, 15 N-tracking metabolomics, microbial ecology, nanoSIMS, nitrogenase expression, Burkholderia

## Abstract

Biological nitrogen fixation by microbial diazotrophs can contribute significantly to nitrogen availability in non-nodulating plant species. In this study of molecular mechanisms and gene expression relating to biological nitrogen fixation, the aerobic nitrogen-fixing endophyte *Burkholderia vietnamiensis,* strain WPB, isolated from *Populus trichocarpa* served as a model for endophyte–poplar interactions*.* Nitrogen-fixing activity was observed to be dynamic on nitrogen-free medium with a subset of colonies growing to form robust, raised globular like structures. Secondary ion mass spectrometry (NanoSIMS) confirmed that N-fixation was uneven within the population. A fluorescent transcriptional reporter (GFP) revealed that the nitrogenase subunit *nifH* is not uniformly expressed across genetically identical colonies of WPB and that only ~11% of the population was actively expressing the *nifH* gene. Higher *nifH* gene expression was observed in clustered cells through monitoring individual bacterial cells using single-molecule fluorescence *in situ* hybridization. Through ^15^N_2_ enrichment, we identified key nitrogenous metabolites and proteins synthesized by WPB and employed targeted metabolomics in active and inactive populations. We cocultivated WPB Pnif-GFP with poplar within a RhizoChip, a synthetic soil habitat, which enabled direct imaging of microbial *nifH* expression within root epidermal cells. We observed that *nifH* expression is localized to the root elongation zone where the strain forms a unique physical interaction with the root cells. This work employed comprehensive experimentation to identify novel mechanisms regulating both biological nitrogen fixation and beneficial plant–endophyte interactions.

## Introduction

Nitrogen (N) is often the most limiting macronutrient for plant growth. In agriculture, this leads to a dependence on N fertilization for maximizing crop yields. The widespread use of synthetic fertilizers not only increases the economic burden to growers, but also has had negative impacts on the environment including eutrophication of waterways and increased levels of atmospheric ammonia and nitrous oxide [1-3]. A promising strategy for improving plant production with minimal environmental impact is to take advantage of the plant–microbe partnerships that have developed naturally in challenging environments. Plant microbiomes, including rhizosphere- and endosphere-associated microbes, have been found to adapt rapidly in order to alleviate host stress [4-10]. Meta-analyses of hundreds of experiments have indicated that these adaptations in microbial relationships can significantly ameliorate the impacts of multiple plant stressors [11, 12]. Therefore, the most effective microbial strains to serve as bio-inoculants under these conditions may be found by tapping into the existing relationships, which nature has already achieved through natural selection in wild plants thriving in challenging environments [13, 14].

Leguminous plants are known to have overcome N limitation through highly efficient symbioses with N-fixing (diazotrophic) rhizobia, but some non-nodulating plant species are also able to colonize low-nutrient sites through close association with a variety of diazotrophic bacteria. Associative N-fixation by endophytes, the free living microorganisms colonizing interior plant tissues, and by other closely associated bacteria can provide significant amounts of N to non-nodulating plant species (reviewed in [15]). Studies using various techniques such as stable isotopes, nitrogenase mutants, total N accumulation, and growth promotion in nutrient-limited conditions have all demonstrated strong evidence for N-fixation of certain diazotrophic strains in a variety of plants including sugarcane [16, 17], Kallar grass [18], wheat [19], rice [20], *Setaria* [21], and in maize [22]. Inoculation of plants by diazotrophic endophytes increased plant growth with reduced fertilizer needs in bioenergy crops including switchgrass [23] as well as poplar [24]. Although the contributions of biological nitrogen fixation (BNF) are clear in these cases, there have also been circumstances where there was no apparent N contribution through BNF and no clear beneficial response to inoculation with diazotrophs [21, 25]. Therefore, to maximize the benefits of these plant–microbe partnerships, a better understanding of the conditions required and underlying mechanisms for effective plant-associated endophytic BNF is needed.

*Burkholderia vietnamiensis* strain WPB was originally isolated in a screen for aerobic diazotrophic endophytes from wild poplar (*Populus*) growing in a low-nutrient, natural riparian setting dominated by cobble and sand [26, 27]. *Burkholderia* are prevalent in the microbiome of wild poplar [28] and, as strain WPB displayed increased nitrogenase activity *in vitro* in response to the addition of air [26], it can serve as a model system for understanding aerobic nitrogen fixation in poplar–endophyte interactions. In this work, we explored the conditions in which nitrogenase activity occurred *in vitro* and *in planta*, and the potential nitrogenous signals or transfer molecules between microbial community members and the host plant.

## Materials and methods

### Bacterial strains, plants, and media

*Burkholderia vietnamiensis* strain WPB [26] was previously isolated from wild *Populus* in the cobble-dominated riparian zone of the Snoqualmie River in Western Washington State. Stocks of the strain maintained at −80°C were used for the experiments. Microbial media used in the study included Nitrogen-Limited Combined Carbon Medium (NL-CCM) [29], NL-MM that is NL-CCM minus mannitol, and rich MG/L medium [30]. Internally sterile *Populus trichocarpa* clone Nisqually-1 [31] plants were maintained on McCown’s Woody Plant Medium with Vitamins (Fisher Scientific, USA), supplemented with 1 μg per liter indole-3-butyric acid rooting hormone. Nitrogen-free medium (NFM) designed for plant experiments with legumes (QubitBiology, Canada) contained the following (μM): KH_2_PO_4_ (257), K_2_HPO_4_ (32), K_2_SO_4_ (500), MgSO4 7H_2_O (243), MgCl_2_ · 6H_2_O (246), CaCl_2_ · H_2_O (748), MnSO_4_ · H_2_O (10), CuSO_4_ · 5H_2_O (1), ZnSO_4_ · H_2_O (1), H_3_BO_3_ (31), NaMoO_4_ · 2 H_2_O (5), CoSO_4_ · 6.5 H_2_O (2), and Fe Sequestrine (33).

### NanoSIMS

Strain WPB was grown on NL-CCM for 3 days. Cell suspensions were made in NFM and adjusted to an optical density (OD_600_) of 0.4. One hundred and fifty microliter was added to 17 ml amber vials containing 6 ml NL-CCM agar. Vials were flushed with argon/oxygen (70/30) and then dosed with either 3.5 ml ^15^N_2_ dinitrogen gas (Sigma) that had been cleaned with hydrochloric acid, to achieve a final concentration of ~20% oxygen, or with 5 ml laboratory air, to approximate equal nitrogen availability. In parallel, a set of WPB vials were prepared for the acetylene reduction assay (ARA), which independently confirmed nitrogen fixation activity of the samples. For NanoSIMS analyses, a total of three natural abundance (NA) and three ^15^N^2^-labeled replicates were prepared. Cells were collected and fixed in 2% paraformaldehyde solution (PFA) at 4°C for 30 min and washed in phosphate-buffered saline (PBS), and shipped to EMSL for analysis.

Quantitative nitrogen isotope images of individual WPB cells were acquired using the NanoSIMS 50 L at the Environmental Molecular Sciences Laboratory at the Pacific Northwest National Laboratory. Approximately 5 × 10^4^ cells from each sample were sorted using Fluorescence-Activated Cell Sorter (FACS) and filtered onto gold-coated, polycarbonate, 0.22 μm filters. Briefly, all NanoSIMS images were acquired using a 16 keV Cs^+^ primary ion beam at 256 × 256 pixel resolution. Secondary ions were accelerated to 8 keV and counted using electron multipliers. For ^15^N/^14^N analyses, 40 × 40 μm areas were pre-sputtered with 1 × 10^15^ ions cm^−2^ prior to collecting ^12^C^14^N^−^ and ^12^C^15^N^−^ secondary ions simultaneously using a 2 pA primary beam with a diameter of ~100 nm. For each sample, six, 35 × 35 μm area images were acquired containing an average of 78 individual WPB cells. A total of 16 consecutive frames were collected for each image using a dwell time of 961 μs px^−1^. The OpenMIMS plugin for Imagej (https://doi.org/10.1371/journal.pone.0030576) was used to align, stack, and correct images pixel by pixel for dead time (44 ns) and QSA (*β* = 0.5). ROI data from individual microbes were exported to a custom spreadsheet for data reduction. Quantitative ^15^N/^14^N analyses were calibrated against an in-house yeast ^15^N isotope reference material during the same analytical session using similar conditions to those used to analyze the bacterial culture samples. Propagation of uncertainties followed standard procedures and included counting statistics of each individual bacterial cell, external uncertainty of 16 individual yeast cells used to calibrate the ^15^N data, the bulk uncertainty of the ^15^N atom% with respect to air (Δ^15^N = +0.35 ± 0.06‰, ±1SD, *n* = 3, air; Jim Moran personal communication).

### Fluctuation localization imaging-based fluorescence *in situ* hybridization

The protocols for fluorescence *in situ* hybridization (FISH) of bacteria, the microscope instrument set up to image single-molecule mRNA FISH, and the data processing for recognizing single mRNA transcripts from images based on single-molecule blinkings (Fluctuation localization imaging-based fluorescence *in situ* hybridization, fliFISH) were previously published in detail [34]. Briefly, WPB bacteria were cultured from cryo stocks in 4 ml NL-CCM with shaking at 240 rpm at 28°C. Cells were then fixed with 4% PFA in PBS at room temperature for 20 min, washed with PBS, and incubated overnight in 50% PBS/50% ethanol at −20°C. FISH probes were designed for the *nifH* subunit gene of nitrogenase. Five segments of mRNA, each 20 nucleotides long, were selected as the FISH targets, with their reverse compliments serving as the FISH probe sequences. The 5′ ends of the FISH probes conjugated with Atto532 dye were purchased from IDTDNA. The FISH probes were hybridized with the WPB cells overnight at 46°C in hybridization buffer (20% formamide, 0.9 M NaCl, 0.02 Tris, 0.01% SDS). The following day, the cells were washed two times in wash buffer (0.9 M NaCl, 0.02 M Tris, 0.01% SDS) and two times with PBS. Cells were imaged with a single-molecule fluorescence microscope (Zeiss Axio Observer) with 100X NA 1.46 oil immersion objection and a fluorescence excitation of 532 nm. The fluorescence from blinking single molecules was recorded by an EMCCD camera (Andor iXon 888), and the events were counted and analyzed to calculate the number of transcripts using MATLAB software. The MATLAB script to count transcripts is available [34].

The fliFISH data were analyzed using R version 4.1.1 (2021-08-10) with the rstatix (v 0.7.1), MASS (v 7.3-54), and emmeans (1.8.5) packages. A chi-square test of independence was performed to assess the association between nitrogen levels and cell association on *nifH* expression, and a Negative Binomial regression model employed to evaluate the effects of cell association and nitrogen status on the mean expression of *nifH* transcripts.

### Transcriptional reporter plasmid construction

The promoter region (270 bp) from the nitrogenase subunit gene *nifH* was cloned pGEM-T-Easy (Promega), and the resulting flanking *Eco*RI sites were used to subclone the promoter into pPROBE-NT-Km, which contains a promoterless GFP [35]. Kanamycin resistant colonies were screened by PCR for the correct orientation of the promoter relative to GFP. The verified plasmid was introduced into wild-type WPB by triparental mating using HB101 (pRK2073) as the helper strain [30]. Carbenicillin, to which WPB is naturally resistant, was used for counter-selection against the DH5α (pPROBE/WPB_P*nif*) donor strain. The resulting transconjugant, WPB (P*nif*-GFP) was streak-purified on MG/L agar containing kanamycin and carbenicillin. Cryo-stocks in glycerol were maintained and used for subsequent experiments.

### WPB P*nif*-GFP *in vitro* assays

WPB (P*nif*-GFP) was grown on NL-CCM containing kanamycin (50 μg/ml) for 2–3 days at 30°C. Initially, cell suspensions were created in NL-CCM broth at varying concentrations from an optical density of 0.1–1.0 and drops of 7, 20, or 100 μl were deposited as dots/pools on NL-CCM agar plates or in wells. Later experiments were at an OD_600_ of 0.1 and were 7 μl pools. To test if active and in-active sectors remained so, a time series experiment was performed. WPB P*nif*-GFP was grown on NL-CCM for 3 days, and a cell suspension was made in NL-MM broth. Seven microliter drops of cell suspension were placed on NL-CCM plates resulting in sectoring of P*nif*-GFP expression. Cell suspensions were then made from active and inactive pools and plated again onto NL-CCM containing kanamycin.

### Flow cytometry

The relative abundance of GFP signal was measured from WPB P*nif*-GFP cells fixed in 4% PFA and processed with a BD Influx FACS (BD Biosciences, San Jose, CA). Using the 488 nm excitation from a Sapphire LP laser (Coherent Inc., Santa Clara, CA) at 100 mW, samples were analyzed using a 70 μm nozzle. Optimization and calibration of the FACS were performed before each analysis using 3 μm Ultra Rainbow Fluorescent Particles (Spherotech, Lake Forest, IL). Forward and side scatter detectors were used to gate out cellular debris and identify the singlet population. The ratio of the two distinct populations of cells, GFP labeled and unlabeled, within a mixed microbial community was identified from 50 000 recorded cells using the Singlet gate. Cells with or without GFP were then collected by FACS.

### ^15^N_2_ time course enrichment for metabolomics and proteomics

WPB was grown in MG/L MM (minus mannitol to aid in cell pelleting) and NL MM broth overnight. Cells were pelleted and washed in NL-CCM, and the optical density (OD_600_) adjusted to 0.4. One hundred and fifty microliter was transferred to 15 ml amber vials with 6 ml NL-CCM agar poured at a slant to increase the surface area. After flushing the vials with argon oxygen, 3.5 ml ^15^N_2_ gas, cleaned with HCl, was injected into the vials. For the time series, samples were collected at time zero and after 3 and 9 days. Laboratory air dosed cells (5 ml) were also collected at each time point. Sample pellets were stored at −80°C until shipment on dry ice to EMSL.

### Sample preparation for metabolomics analysis

For extraction, 1 mL of cold (−20°C) chloroform:methanol mix (prepared 2:1 (v/v)) was pipetted into chloroform-compatible 2 mL microcentrifuge tubes kept on ice. Then, 200 μl of the sample homogenate in water was added to the tube and vigorously vortexed. The sample was then placed in the ice block for 5 min, vortexed for 1 min, followed by centrifugation at 10 000 × *g* for 10 min at 4°C. The upper water-soluble metabolite phase and lower lipid soluble phase were collected into the same glass vial for metabolomics analysis. A wash of the pellet with 500 μl of methanol was done, followed by centrifugation and addition of the methanol portion to the vials containing the upper and lower phase already collected. The extracts were completely dried in a speed vacuum concentrator and then capped and stored dry at −20°C until analysis. The pellet was lightly dried in a fume hood and then stored at −80°C until protein digestion.

Dried extracts were chemically derivatized as previously reported [36]. Briefly, the extracted metabolites were derivatized by methoxyamination and trimethylsilylation, and then the samples were analyzed by gas chromatography–mass spectrometer (GC–MS). Samples were run in two instruments: an Agilent GC 7890A coupled with a single quadrupole MSD 5975C (Agilent Technologies) for low-resolution measurements and a Thermo Q-Exactive GC–MS system (Thermo Fisher Scientific) for the high-resolution MS. Each of the GC instruments was equipped with an HP-5MS column (30 m × 0.25 mm × 0.25 μm; Agilent Technologies, Santa Clara, CA). One microliter of derivatized sample was injected into a splitless port at constant temperature of 250°C. The GC temperature gradient started at 60°C, with a hold of temperature for 1 min after injection, followed by increase to 325°C at a rate of 10°C/min and a 5-min hold at this temperature. A fatty acid methyl ester standard mix (C8–28) (Sigma-Aldrich) was analyzed in parallel as standard for retention time calibration.

### ^15^N metabolomics data analysis

GC–MS data files were processed by three different software programs publicly available for analysis of isotope enriched metabolites: MIA [37], MIAMI [38], and DExSI [39]. During the analyses using MIA and MIAMI, ^15^N-enriched metabolites were screened and selected as untargeted manner using PNNL-in-house metabolite library for identification. Subsequently, the ^15^N-enriched peaks were further analyzed by DExSI software with retention time and chemical formula information of each metabolite to correct for mass isotopomers because of NAs and chemical derivatization. All the values and graphical data were generated from DExSI.

### ^15^N proteomics data analysis

#### Proteomic sample preparation and mass spectrometry

Protein pellets obtained from extractions were solubilized by overnight incubation at 4°C in Denaturing Protein Solubilization buffer (7 M urea, 4% CHAPS, 10 mM TCEP in 50 mM NH_4_HCO_3_ then extracted by sonication (Bransonic 1800 Ultrasonic Bath CPX1800H), incubation at 60°C for 30 min, repeat of sonication, and centrifugation (10 min, 5000 × *g*, at 4°C). The Coomassie Protein Plus assay was used for protein quantification. Iodoacetamide (final 20 mM) was added to alkylate cysteines. Ammonium bicarbonate (final 50 mM) solution and calcium chloride (final 2 mM) solution were added to dilute the original Protein Solubilization buffer 8-fold and then the sample was digested overnight at room temperature with trypsin (1:50, enzyme:protein ratio).

Sample digests were cleaned using SCX columns (MilliporeSigma Supelclean LC-SCX SPE Tube 100 mg CAS 504920) following manufacturer’s recommended protocol with two additional washes of 70% Methanol in 0.1%TFA (2 mL) before final elution. Samples were concentrated in to 50 μl in a vacuum concentrator (Savant Model: SC250EXP) and then diluted to 100 μl with nanopure water. Bicinchoninic acid assays were conducted to determine peptide yields.

Tryptic peptide samples were diluted to 0.1 μg/μl in nanopure water for analysis by LC–MS/MS. Five hundred nanogram of each peptide sample was separated on an in-house packed reversed-phase capillary column (4 cm × 150 μm i.d.) with 5 μm Jupiter C18 particles at room temperature using a nanoAcquity HPLC system (Waters Corporation, Milford, MA). The elution was carried out at 300 nL/min with a gradient of 0.1% formic acid in water (mobile phase A) to 0.1% formic acid in acetonitrile (mobile phase B) (0–2 min 1% B; 2–20 min 8% B; 20–75 min 12% B; 75–97 min 30% B; 97–100 min 45% B; 100–105 95% B; 105–110 min 95% B; 110–140 min 1% B).

Mass spectra were collected from the resulting eluent on a QExactive Plus hybrid quadrupole-Orbitrap mass spectrometer (Thermo Fisher Scientific), operating in high-resolution, higher collision energy dissociation, and data-dependent acquisition modes with the following settings: full MS (AGC: 3 × 10^6^; resolution: 70 000; *m/z* range: 300–1800; maximum ion time, 20 ms) and MS/MS (AGC: 1 × 10^5^; resolution: 17 500; *m/z* range: 200–2000; maximum ion time: 50 ms; TopN: 12; isolation width: 1.5 *m/z*; dynamic exclusion: 30.0 s; collision energy: NCE 30). Data were acquired for 2 h, resulting in 53 936–60 659 scans generated per sample.

#### Peptide identification

The resulting raw data files were converted to centroided mzML format using ProteoWizard msConvert [33] with peak picking on MS1 and MS2 spectra. MSGFPlus [40] was used to search resulting spectra against a targeted protein database of 5962 translated proteins from the *B. vietnamiensis* WPB genome (JGI IMG Genome ID 2615840639), appended with 116 common laboratory contaminants from the cRAP database (https://www.thegpm.org/crap/). Search parameters allowed for 10 ppm precursor mass tolerance, variable modifications up to a maximum of three per peptide, including methionine oxidation and incorporation of heavy nitrogen (^15^N), and up to one missed trypsin cleavage site. Cysteine carbamidomethylation was included as a static modification. The resulting peptide-spectrum matches were controlled to a false discovery rate of 1% at the peptide level using the target-decoy method within percolator [41].

#### Peptide atom% ^15^N determination

Peptide search results were processed through the classified Peptide Isotope Enrichment (cPIE) workflow [42] to determine peptide-specific isotope incorporation (atom% ^15^N). This approach involves extracting MS1 intensities to calculate relative abundances of all isotopologue species for each identified peptide, correcting enrichment estimates for potential contaminating signal from NA isotopes, and filtering results through a logit classifier trained on bacterial culture data of known isotopic enrichment to exclude likely false-positive results, all implemented in R v4.0.5 (R Core Team, 2017). Preliminary isotopic enrichment estimates were successfully generated for 36 863 peptides, of which 20 592 peptides passed the classifier.

#### Peptide functional annotations

Peptide annotations were derived from annotations of associated protein sequences from the *B. vietnamiensis* WPB genome. All peptides were searched against the translated WPB protein sequences using the Aho-Corasick algorithm implemented in the AhoCorasickTrie R package v0.1.2 (https://CRAN.R-project.org/package=AhoCorasickTrie) to identify all possible parent proteins for each peptide. Annotations from multiple possible parent proteins per peptide were consolidated by assigning the mode of all KEGG Orthology identifiers as the predicted peptide function.

#### Targeted analysis of metabolite pathways

Parallel ^15^N-metabolomics analysis identified 15 metabolites of interest that were isotopically enriched during the incubation. KEGG pathway maps associated with these metabolites of interest were compiled into a list of proteins for targeted analysis (map00220, 00250, 00260, 00290, 00300, 00330, 00350, 00360, 00400, 00410, 00480, 00563, 00564, 00600, 00650, 00760, 00960, 02024), as well as any proteins involved in nitrogen metabolism (map00910). Of the 20 592 peptides that passed the cPIE classifier with isotopic enrichment estimates, 3176 were associated with these proteins of interest. Extracted ion chromatograms were manually curated to exclude poorly assigned isotope envelopes (e.g. because of noisy peaks or contamination from co-eluting peptides), reducing the dataset to 2684 peptides. Subsequent analysis focused on a subset of 1621 of these peptides that were observed in at least two of six biological replicates.

### RhizoChip methods

RhizoChips, designed to replicate the pore space structure of soil, were fabricated from polydimethylsiloxane (10:1 wt/wt, PDMS base to curing agent; Sylgard 184 Dow Corning) and bonded to a glass coverslip using oxygen plasma as described [43]. A pipette tip was trimmed and inserted into the RhizoChip inlet to provide support for the stem of the poplar cutting. The entire RhizoChip was then autoclaved sterilized and then liquid media (1/4x Hoaglands with or without N) was used to saturate the RhizoChip pore space (total volume of ~246 μL). *Populus trichocarpa* cuttings were prepared for the RhizoChip experiments by cutting shoot apical meristem tissue from an axenically grown mother plant and transferring the cutting to Magenta box with Woody Plant Media Agar amended with IBA plant hormone to encourage rooting. Cuttings were kept at 22°C in a growth chamber with 18 h/6 h light/dark cycle for ~1 week until roots had begun to develop but were <1 cm in length. At this point, a rooted cutting was transferred to the inlet of the RhizoChip. RhizoChips were then placed in a transparent box to maintain axenic growing conditions and returned to the growth chamber. For plants that were inoculated with endophyte, *B. vietnamiensis* WPB P*nif-GFP* was grown overnight in R2A media then spun down and resuspended to an optical density (OD_600_) of 0.5 in the appropriate 1/4x Hoaglands media. Once roots had begun to grow into the RhizoChip pore space, plants in the endophyte treatment group received a dose of WPB culture by pipetting the liquid inoculum into the ports around the RhizoChip. After at least 4 days of co-culture with the WPB, the RhizoChips were imaged on a Leica confocal microscope with a 63x oil objective using brightfield, GFP and BFP filters. The roots with WPB were also imaged under water by a lattice lightsheet microscope (Intelligent Imaging Innovations). A 488 nm wavelength laser was used to excite the fluorescence from GFP in WPB and the autofluorescence from the plant root cell walls.

## Results

### Nitrogenase gene expression is dynamic

**Figure 1:**
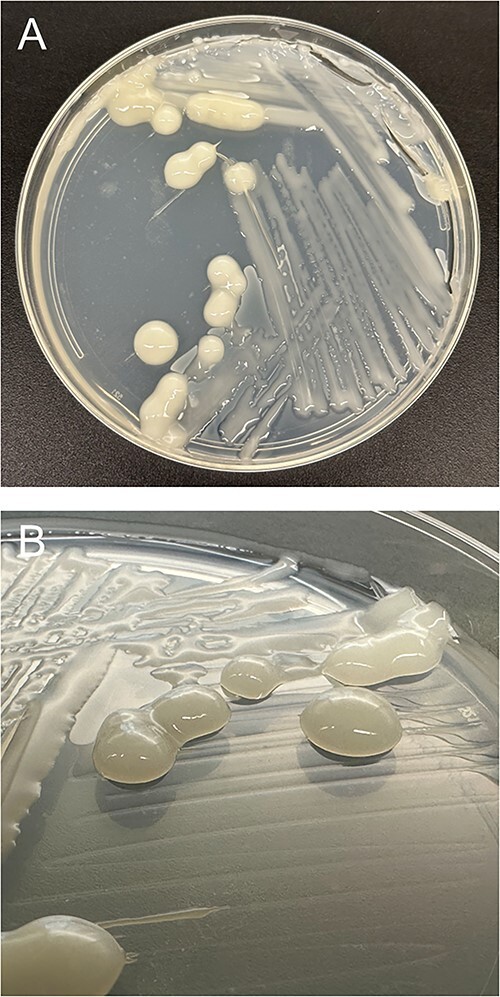
WPB on NL-CCM plates displays heterogeneous growth patterns. (**A**) Top down view of plate shows large colonies intermixed with lawns of less vigorous growth. (**B**) Oblique view of plate shows raised, globular-like growth of the larger colonies.

**Figure 2:**
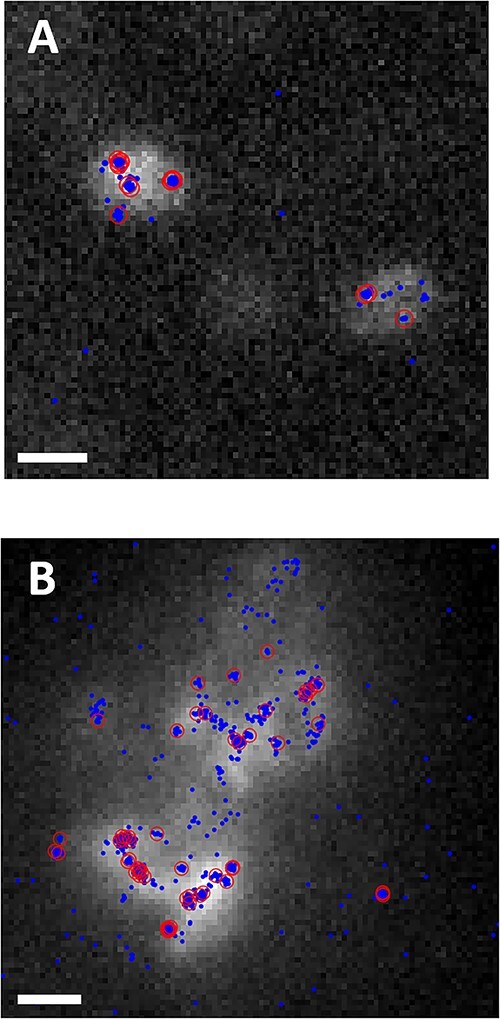
The fluorescence images of WPB cells and direct quantification of *nifH* mRNA transcripts in single cells in a population. Closed dots indicate fluorescence blinking events. Open circles indicate actual transcript copies as determined based on blinking events per unit time. The scale bar is 1 μm. (**A**) Isolated cells; (**B**) aggregated cells.

When grown on nitrogen-free agar media, colonies of strain WPB differed in size, with some forming very large, globular-like colonies compared with the more moderate growth of others (Fig. 1). To quantify diazotrophic activity at the level of transcription within individual cells, direct counting of mRNA transcripts was performed. fliFISH [34] was used to directly quantify *nifH* transcripts in single bacterial cells (Fig. 2). Although both cell association (aggregate vs. planktonic) and nitrogen status (replete vs. limited) influenced *nifH* expression in the endophyte WPB (*X*^2^_df = 1_ = 29.26, *P* << .001), cell association had a greater influence on *nifH* expression than nitrogen status. Aggregated cells produced, on average, 30 times more *nifH* transcripts than the planktonic cells (19.5 vs. 0.69) (Table 1), suggesting nitrogenase gene regulation by cell density, quorum sensing (QS), or the structural environment.

**Table 1:** Mean *nif*H transcription levels by cell association and nitrogen status. Cell association had a greater influence on nifH expression than nitrogen status (*β* = −3.32, *z* = −20.58, *P* << .0001). Nitrogen-limited had a positive, but nonsignificant, effect on *nif*H expression (*β* = 0.17, *z* = 1.22, *P* = .22). Negative Binomial regression was used to estimate effects of cell association and nitrogen status on *nif*H transcript expression.

	**Transcripts**		
**Nitrogen status**	***n***	**Mean ± SD**	**95% CI**
**Aggregate**			
N Replete	36	14.53 ± 10.56	10.93–18.13
N Limited	28	25.82 ± 16.71	19.32–32.32
**Planktonic**			
N Replete	49	1.02 ± 0.75	0.82–1.22
N Limited	45	0.33 ± 0.71	0.13–0.53

Though not fully repressed, the aggregated cells produced higher *nifH* transcript counts (nearly 2-fold) when grown under nitrogen-limited conditions compared with nitrogen-replete conditions. The planktonic cells grown under nitrogen-replete conditions expressed *nifH* at much higher levels, producing 3-fold more *nifH* transcripts, on average, than planktonic cells grown under nitrogen-limited conditions. These data indicate that the expression of the nitrogenase gene in endophyte strain WPB is not fully repressed by the presence of nitrogenous compounds, both in isolation and in community, a trait that could benefit the plant during microbial symbiosis.

**Figure 3:**
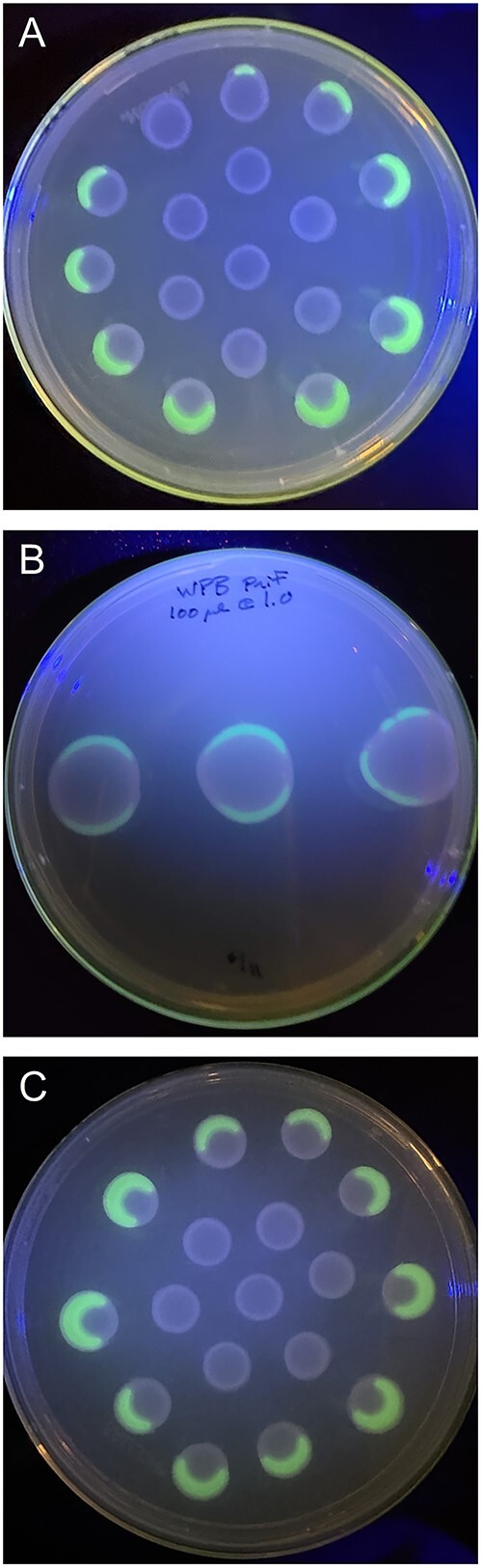
Examples of spatial distribution of nitrogenase gene expression in WPB P*nif*-GFP grown on nitrogen limited agar. Pools of cell suspensions across multiple experiments display heterogeneous expression patterns. (**A**) Pools of 7 μl from an OD_600_ 0.4 cell suspension; (**B**) pools of 100 μl from an OD_600_ 1.0 cell suspension.

A reporter fusion between the promoter of the nitrogenase subunit gene *nifH* and green fluorescent protein (GFP) was introduced into the strain, hereafter termed WPB (P*nif*-GFP). Cell suspensions plated as pools onto NFM containing antibiotic selection for the plasmid gave rise to distinct spatial distribution of nitrogenase gene expression. Though the pools of cells were identical (all from the same cell suspension), some were inactive, showing no nitrogenase promoter-GFP fusion activity, whereas others were fully active. Furthermore, many of the pools had defined sectors of *nifH* expression activity. Generally, but not always, the pools nearest the outer edges of the petri dishes were most active whereas the cell suspensions plated near the center were generally inactive (Fig. 3). Of these active pools, the outer periphery was usually seen to have the most nitrogenase promoter activity. Over time, the inactive areas remained inactive, whereas the *nifH*-expressing zones continued to express GFP (Supplementary Fig. 1). Cell suspensions made from the active and inactive areas replated onto N-free medium resulted in the reformation of expression sectoring based largely on location, not on whether or not the cell suspensions originated from active or inactive sectors (Supplementary Fig. 1), indicating that there are strong environmental influences on nitrogenase gene expression.

To quantify nitrogenase expression variability within the population, fluorescing and non-fluorescing WPB cells with the GFP reporter were sorted by FACS. WPB (Pnif-GFP) cells with active GFP represented only 11.5% of the cells (Supplementary Fig. 2).

### Nitrogen fixation activity is variable within the population

**Figure 4:**
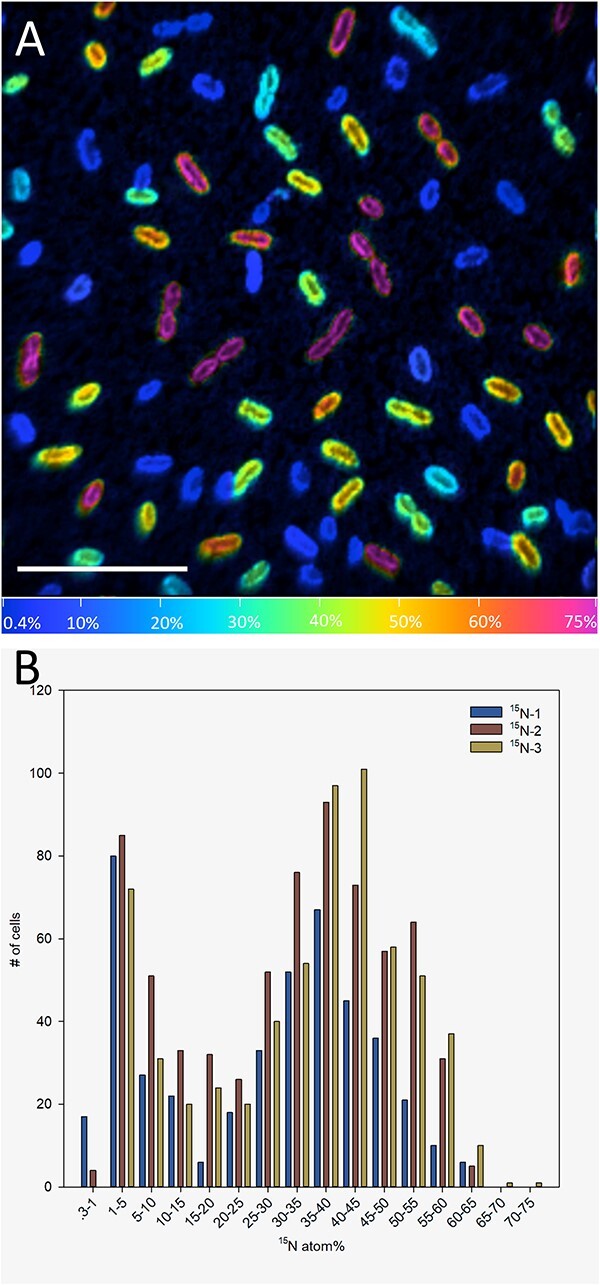
(**A**) Representative hue saturation intensity (HSI) ion image of a^15^N-labeled WPB culture. Hues represent ^15^N atom% values of individual cells. Incorporation is heterogeneous in pure culture of WPB. (**B**) Histogram representing NanoSIMS analyses of three biological replicates of the *in vitro*^15^N_2_ fixation experiment. Bar height represents number of WPB cells in each enrichment class for each replicate. NanoSIMS was used to produce individual ^15^N atom% estimates from six separate images for each replicate. Cell numbers ranged from 440 to 682 individual cells and enrichments of 0.6–70.3 atom% between the three replicates.

Since these transcriptional reporter results are not direct assessments of nitrogenase gene expression or nitrogen fixation, we used secondary ion mass spectrometry (NanoSIMS) to test identical WPB populations exposed to either laboratory air or to ^15^N_2_ gas (Fig. 4). All of the ^15^N_2_-dosed cells were significantly enriched in fixed ^15^N with respect to air controls. However, the population exhibited enrichment over a wide range, from 0.58 to 70.29 atom percent ^15^N, following a bimodal distribution of ^15^N enrichment in all three replicates (Supplementary Table 1). These direct determinations of nitrogen fixation further illustrate that the dynamic nature of nitrogenase gene expression in strain WPB extends to realized biological activity.

### Fixed N is directed toward amino acids and polyamine biosynthesis

**Figure 5:**
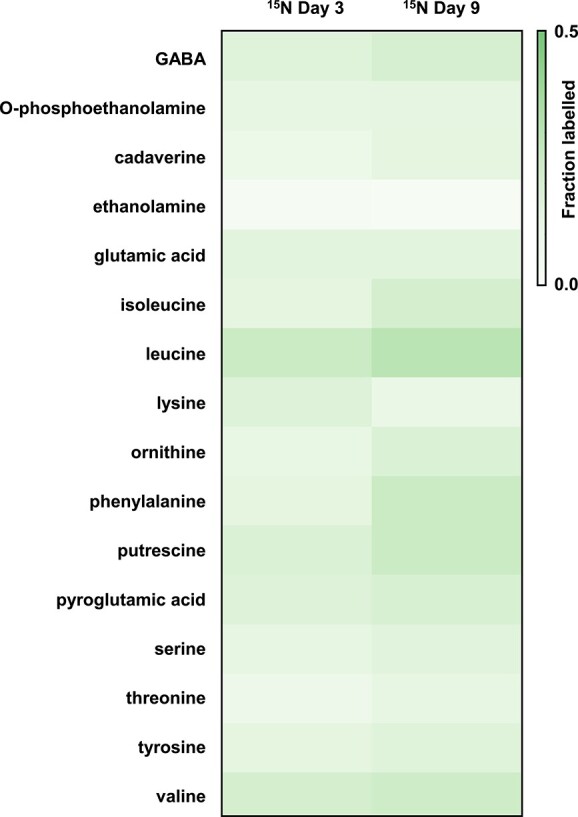
Heatmap analysis of isotope enriched nitrogenous metabolites from *B. vietnamiensis* WPB. ^15^N-enriched metabolites were plotted based on the labeled fractions. Most of the nitrogenous metabolites were labeled under the ^15^N condition. Leucine was heavily co-eluted with other metabolite peaks so the shown background noise is high.

**Figure 6:**
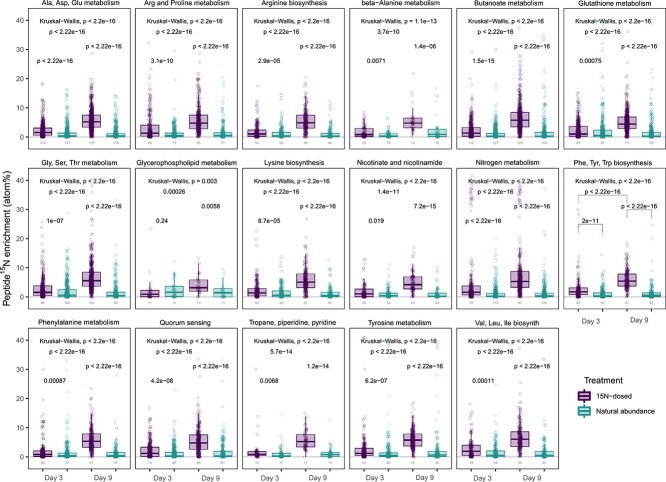
Atom% ^15^N enrichment of peptides from WPB cultures collected after 3 or 9 days of incubation with either ^15^N_2_ or ^14^N_2_, grouped by KEGG pathway. Datapoints represent individual peptides. Numbers at the bottom indicate the number of peptides detected in each treatment and time point. Overall, peptides associated with each pathway were significantly enriched in ^15^N_2_ vs. ^14^N_2_ treatments, as well as between 3 and 9 days of incubation (significance of pairwise comparisons shown on plots from Wilcoxon rank sum tests with Benjamini–Hochberg corrections for multiple testing), although individual peptide enrichment values varied. The *y*-axis is truncated to better show inter-treatment comparisons. Note that some pathway names have been abbreviated for visualization (“Nicotinate and nicotinamide metabolism” to “Nicotinate and nicotinamide”; “Tropane, piperidine and pyridine alkaloid biosynthesis” to “Tropane, piperidine, pyridine”).

WPB cells were incubated with ^15^N_2_ to identify key nitrogenous metabolites and proteins that are synthesized by WPB with ^15^N_2_ as the sole nitrogen source. Global metabolomics and proteomics revealed 10 key metabolites enriched in ^15^N that were also associated with metabolic pathways that had peptides enriched in ^15^N. Gamma-aminobutyric acid (GABA), a nonprotein amino acid, several polyamines including putrescine and cadaverine, and seven amino acids were among the signature metabolites enriched in ^15^N (Fig. 5). Peptides associated with pathways for biosynthesis of valine, leucine, and isoleucine as well as tyrosine and butanoate metabolism had the highest median enrichment estimates (~6 atom% ^15^N). Compared with NA controls, all of these detected pathways showed significant enrichment in peptides over the 9-day experiment (Fig. 6) (pairwise Wilcox on rank sum test with Benjamini–Hochberg corrections for multiple testing; *P* < .05). These protein stable isotope probing (SIP) results confirm that WPB directs freshly fixed N_2_ toward metabolic pathways producing polyamines and amino acids. In addition, 10 ^15^N-enriched peptides were annotated as being in QS-associated pathways. These included FlhD, a flagellar transcriptional activator; LivK, a branched-chain amino acid transport system substrate-binding protein; ABC.SP.S, a putative spermidine/putrescine transport system substrate-binding protein; QseC, a sensor histidine kinase; and SdiA, a quorum-sensing system regulator.

### *nifH* expressing sectors produced more polyamines

**Figure 7:**
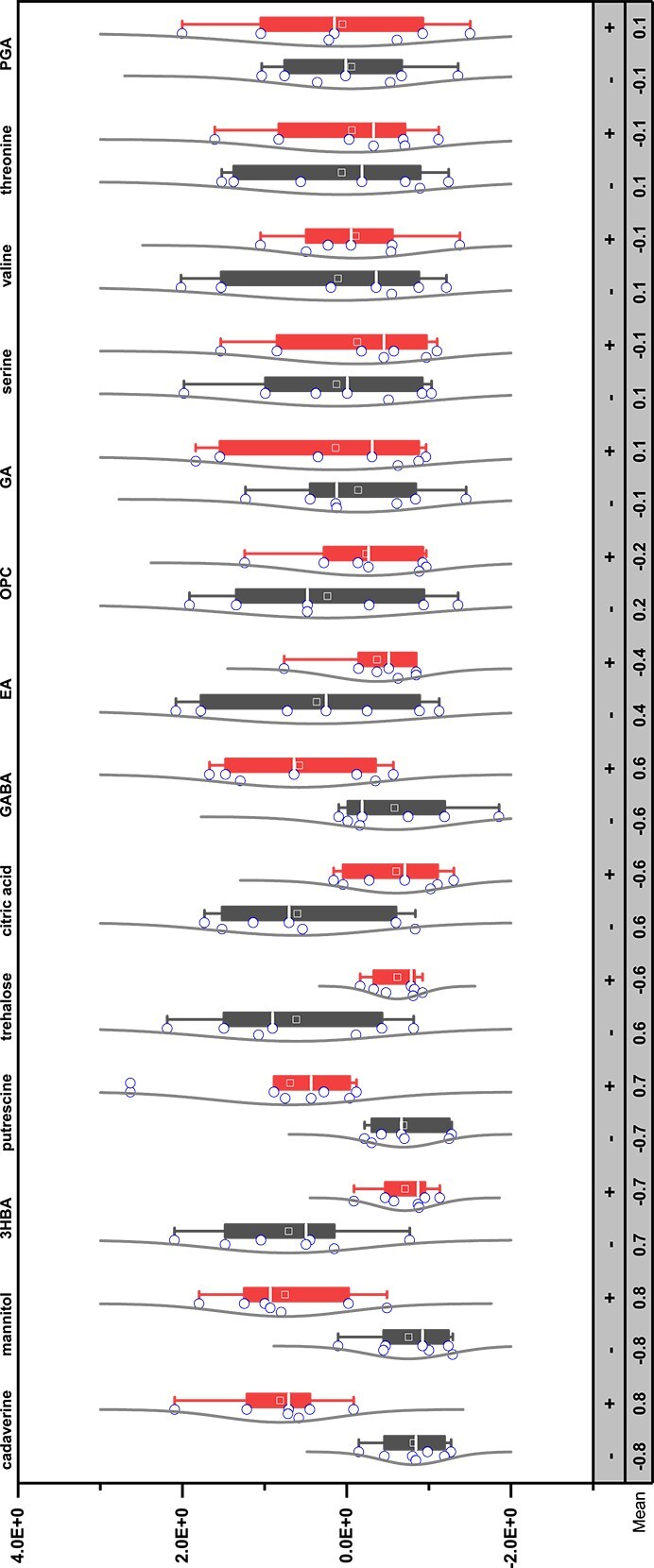
Selected metabolites from the sectoring experiment with *nifh* gene expression. The abundance profiles of selected metabolites from the biomass with the expression of nifh gene (+ symbol) and without (− symbol). Polyamines (cadaverine and putrescine) were strongly correlated with the expression of the gene, and other metabolites were positively or negatively related with the expression. Peak area values of individual metabolites were normalized by corresponding dried biomass weight first, and then followed by *z*-score transformation. Abbreviation: 3HBA, 3-hydroxybutyric acid; EA, ethanol amine; OPC, o-phosphocolamine; GA, glutamic acid; PGA, pyroglutamic acid.

To test if the differential expression of GFP in the WPB P*nif*-GFP pools on N-free medium was truly indicative of nitrogen fixation rather than only differences in nitrogenase gene expression, we assessed the *nifH* active cells from active and inactive sectors for the presence of the key nitrogenous metabolites (Fig. 7; Table 2). The cells that were strongly expressing the nitrogenase promoter reporter contained more putrescine and cadaverine than the *nifH*-inactive cells of the same cell suspension pool. The cells of the *nifH*-inactive sectors produced more trehalose and citric acid than the cells in the nitrogenase active sectors. Putrescine, cadaverine, and GABA were detected in the inactive zones but at significantly lower levels, potentially because of diffusion or “sharing” of the fixed N. These data support the interpretation that the different sectors do have different dominant metabolic activities, and allude to the possibility that inactive cells support the activity of the nitrogen-fixing cells perhaps through providing energy sources.

**Table 2:** Average metabolite abundance from colony sectors (normalized by biomass) with (*n* = 7) and without (*n* = 7) visible *nif*H reporter expression, ^a^ = identified in global metabolomics in ^15^N study but below limit of detection here, ^b^ = significantly different between sectors with and without *nif*H expression.

**Metabolites**	**nifH+**	**nifH−**	***P*-value**
Cadavarine	128 685	50 729	.3579^b^
Mannitol	33 220 258	11 385 608	.4196^b^
3-Hydroxybutanoic acid	157 854	521 756	.1028^b^
Putrescine	1 125 966	560 994	.5668^b^
Trehalose	5 164 252	14 509 326	.0826^b^
Citric acid	4775	8974	.2211^b^
GABA	6714	3519	.9346^b^
Ethanolamine	84 767	110 068	.0505
O-phosphocolamine	13 034	16 589	.6490
Glutamic acid	137 783	118 484	.8843
Serine	34 933	41 539	.2834
Valine	7218	8018	.2558
Threonine	12 380	13 133	.2651
Pyroglutamic acid	63 170	59 859	.5217
Tyrosine	^a^	^a^	N/A
Ornithine	^a^	^a^	N/A
Lysine	^a^	^a^	N/A
Isoleucine	^a^	^a^	N/A

### Nitrogenase expression in association with poplar roots

**Figure 8:**
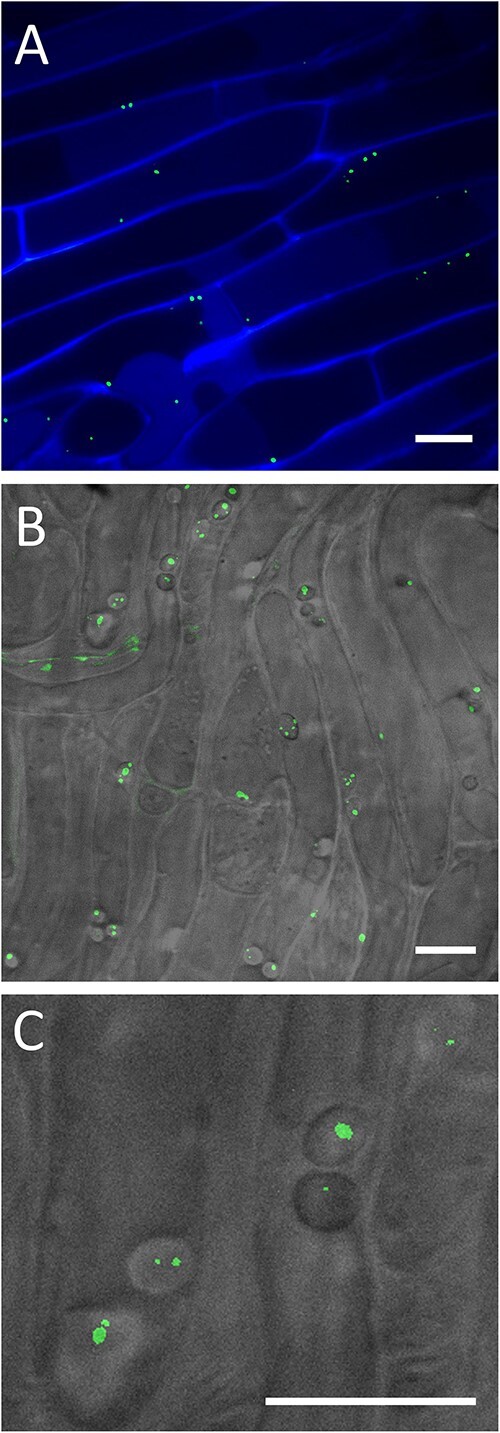
Confocal images of *P. Trichocarpa* roots growing inside a RhizoChip show (**A**) that WPB cells (fluorescent dots) express the *nifH* gene inside of root epidermal cells, and (**B**, **C**) fluorescence from *nif*H-expressing WPB cells is sometimes localized to spherical structures (referred to as nitrosomes in this work) roughly 3–5 μm in size inside of epidermal cells in the root elongation zone.

We cocultivated WPB with the P*nif*-GFP reporter with poplar (*P. trichocarpa*) *in vitro* using RhizoChips, a synthetic soil habitat [43] that enabled us to image the expression of microbial *nifH* within root epidermal cells. The nitrogenase gene was expressed while in association with the host plant, demonstrating that endophytic conditions are conducive to nitrogen fixation for this aerobic diazotroph (Fig. 8, Supplementary Fig. 4, and Supplementary Video). The WPB cells expressing the *nifH* reporter were visible within the boundaries of poplar root epidermal cells, primarily within the elongation zone of the roots. The *nifH* gene was expressed regardless of whether there was added N in the surrounding medium, a regulatory condition not uncommon in symbiotic plant–microbe interactions, and consistent with the *in vitro* data from WPB alone. Compared with a no-bacteria control, poplar roots inoculated with WPB displayed small (3–5 micron) spherical structures within the epidermis, suggesting a potential specific physical interaction with the diazotrophic bacteria. The nitrogenase gene reporter fusion was active in these structures, so we termed them “nitrosomes” as potential sites for active nitrogen fixation (Fig. 8). Future research will explore the dynamics of these structures and their function in the poplar–diazotrophic endophyte interaction.

## Discussion

Nitrogen fixation in wild *Populus* plants is highly variable with high and low activities in adjacent sections of the same plant sample [44]. Unlike symbioses in the legume–*Rhizobium* interaction where the active diazotrophs are sequestered into root nodules, endophytic nitrogen-fixation could occur anywhere within non-nodulating plants as long as the required microbial population and environmental conditions are met. To begin to understand the complexity and molecular mechanisms of endophytic nitrogen fixation, we characterized a single, representative aerobic endophytic strain from wild poplar *in vitro* and *in planta* using a suite of complementary multi-omics, chemical, and imaging approaches.

*Burkholderia* species are common members of the plant microbiome and are capable of free-living, associative, and symbiotic N-fixation [45-47]. The Burkholderiaceae was one of the most common families of the core microbiome of wild *Populus* [28]. *Burkholderia vietnamiensis*, initially isolated from rice, was the first species of *Burkholderia* identified to be diazotrophic [48] and has since been isolated from various traditional rice cultivars [49] as well as from maize and coffee plants in Mexico [47]. The addition of *Burkholderia* strains from rice in India increased yields, with 42% of the nitrogen attributed to BNF [45]. *Burkholderia vietnamiensis* from rice in Brazil increased grain yields up to 29% and resulted in grains with higher protein content [50]. *Burkholderia vietnamiensis* strain WPB was isolated from the branches of wild *Populus* in a natural riparian setting in the Pacific Northwest. Addition of WPB to grasses also increased plant growth and total nitrogen content [26]. However, the details of the underlying molecular mechanisms of these endophyte–plant interactions are not yet known.

As with endophytic nitrogen fixation within wild plants, we found nitrogenase activity to be highly dynamic even within the microbial population of a single strain grown *in vitro* (Fig. 3). Overall, only about 10% of the WPB cells in a population were actively expressing the nitrogenase gene (Supplementary Fig. 2). The small percentage of N-fixing cells in populations has been seen in other systems such as cyanobacterial mats where only a small subset of the non-heterocyst cyanobacterial cells capable of N-fixation were actively fixing N [51]. In our study, under nitrogen limited conditions in liquid media, WPB cells in aggregates expressed *nifH* 30x more than did planktonic cells (Table 1; Fig. 2). A possible explanation for this difference would be quorum sensing, such that nitrogen fixation only occurs at higher cell densities. This could be a first step in bringing cells together to form a broader functioning diazotrophic community. QS is highly prevalent in poplar endophytes, with nearly half of the 39 sequenced root endophyte genomes containing the *luxI* gene family associated with QS [52]. It has also been shown that QS induced phenotypic switching in some strains of rhizospheric *Pseudomonas* can affect biofilm formation and plant–host interactions [53]. It is possible that a similar phenomenon is occurring in WPB cells, where aggregated cells are using QS to alter gene pathways related to nitrogen fixation. Here, proteins involved in QS pathways were indeed significantly enriched in the ^15^N treatment, providing further support that these pathways are active during N-fixation (Fig. 6). One of the peptides annotated as being associated with QS pathways was ABC.SP.A, a putative spermidine/putrescine transport system ATP-binding protein, and another was LivK, an amino acid transport system substrate binding protein. It may be that WPB does not export the products of nitrogen fixation until a sufficient population is present. In future, we will test QS involvement by creating mutants in identified QS genes to determine if their absence has a significant impact on population-wide interactions.

Nitrogen fixation may be an activity requiring differentiated metabolic roles because of the high energy demand. In addition to the 20–30 ATP required to reduce each N_2_ molecule, cells also need to synthesize large amounts of the nitrogenase enzyme, up to 20% of the total cell protein [54]. We propose that the high energy demand of N-fixation requires cooperation such that the non-N-fixing cells of a population may provide the sugars and energy to support the activity of the minor actively N_2_-fixing population. Trehalose and citric acid, found in higher levels in the inactive sectors of the WPB pools (Fig. 7), could be energy sources supplied by the inactive cells to the nitrogen-fixing cells, or they may help with creating a microenvironment suitable for nitrogen fixation. Trehalose, a disaccharide of glucose, is common in a variety of organisms as an energy and carbon source but it can also serve to protect proteins from inactivation during stresses including oxidation [55]. Considering that the nitrogenase enzyme is acutely sensitive to inactivation by oxygen, trehalose may assist in protecting the enzyme through the conversion of trehalose to exopolysaccharides that can encase the nitrogen-fixing cells in a biofilm sac [56]. Citric acid is a key component of the Krebs cycle from which ATP and reducing power are generated. The increased levels of both trehalose and citric acid in the non-fixing cells suggest cooperation in the microbial community to promote the nitrogenase activity of the minority of the population.

From visual observations of wild-type WPB and WPB containing the P*nif*-GFP reporter on nitrogen-limited plates, it appeared that environmental effects may be governing regulation of nitrogen fixation in this context (Fig. 3), compared with in the liquid cultures. The GFP reporter for nitrogenase gene expression indicated a strong spatial distribution of expression even though each population of cells in the plated pools would be essentially genetically identical with only the cell’s location on the plate varying. The cells that were expressing the *nifH*-GFP reporter formed a 3D structure (Fig. 1), appearing to have more growth; however, those cells could also be producing more extracellular polymeric substances (EPS) without increasing cell number [57] in order to protect the enzyme from oxidation [56]. In our previous work, WPB nitrogenase activity, as indicated by the ARA, increased when additional air was added [26], likely providing the oxygen needed for the high ATP demands of nitrogen fixation. This correlates to the sectoring we observed, with N-fixation occurring on the outer periphery of plates (Fig. 3). Therefore, oxygen status is one parameter that may regulate the differential fixation of nitrogen within a community of WPB cells. The nitrogenase enzyme is acutely sensitive to oxygen yet the nitrogenase activity is high energy demanding, such that some free-living diazotrophs have internal mechanisms to protect the enzyme including respiratory control, cytochromes, triterpenes, and physical partitioning [32, 58, 59]. External mechanisms of protection, including the formation of biofilm sacs around active diazotrophs, have been observed in several nitrogen-fixing strains [56].

A common regulator of nitrogenase gene expression is nitrogen availability. However, with strain WPB, cell association (planktonic or aggregated) had a greater influence on *nifH* expression than did nitrogen status (Table 1; Fig. 2). Whereas in many diazotrophic species, nitrogenase gene expression is strongly repressed when nitrogen is available, there are some systems, particularly in mutualists, where nitrogenase is not repressed [54]. This lack of repression may be a characteristic of symbionts where sharing of excess nitrogen is a requirement of the trade for fixed carbon from the plant.

The form of N transferred from active diazotrophic endophytes to the rest of the microbial community or to the host plant is generally unknown. In this work, we identified new “signature” molecules that may represent the initial nitrogenous compounds produced by strain WPB that can be assessed in inoculated plants and serve as potential indicators of active endophytic nitrogen fixation. Through ^15^N tracer experiments, we identified the key nitrogenous compounds formed in WPB to be polyamines and amino acids (Fig. 5). Polyamines occur ubiquitously in microorganisms, plants, and animals. In plants, polyamines are associated with tissues that are actively growing and can be involved in the plant stress response [60-62]. Other significantly labeled metabolites we detected included putrescine and GABA. Putrescine can be substituted for nitrates in tissue culture-grown plants and suspension cultures [61, 63]. The nonprotein amino acid, GABA, is also widely found in nature and can serve as an N storage molecule as well as a signal molecule in plants [64-66]. Our complementary protein SIP results indicated that WPB was actively synthesizing the proteins associated with metabolite synthesis (Fig. 6) in addition to synthesizing the metabolites themselves. In line with our results, poplar inoculated with diazotrophic *Paenibacillus* had higher levels of asparagine and urea, whereas sugars and organic acids decreased [67]. The amino acid asparagine is one of the major transport amino acids in poplar and the main export form of nitrogen in *Medicago* nodules [67]. Thus our work provides further support for the likely important role of amino acids as a currency of nitrogen exchange in endophyte–plant relationships.

WPB expressed nitrogenase when in association with poplar roots, indicating that the appropriate environmental criteria are present (Supplementary Video 1). Plant signals or metabolism may therefore be involved in nitrogenase regulation. “Orphan” or solo *lux*R homologs were previously identified in poplar endophytes such that, instead of the bacterium producing the required signal molecule received by the LuxR partner for QS, the signal molecule may have come from other members of the plant microbiota or from the plant host itself [52]. Though in the case of strain WPB that fixes nitrogen both in a free-living state and in association with the plant, these plant signals may be less important or may have increased the number of active cells. Further research is needed to compare the diazotrophic activities *in vitro* and *in planta*.

Within the plant cells, WPB P*nif*-GFP was expressed in vesicle-like structures we termed “nitrosomes” that were only present in the plant cells if WPB was present, and we have not seen such structures with pure WPB cells grown *in vitro* (Fig. 8). It has been reported that free-living diazotrophs form large oval biofilm sacs the authors termed “cysts” containing two to eight cells per sac [56]. Diazotrophic *Pseudomonas stutzeri* and *Azospirillum brasilense* formed these cysts comprised primarily of EPS (exopolymeric substances), especially exopolysaccharides, in response to low nitrogen, higher than 1% oxygen, and a sufficient carbon source [56]. In the legume–*Rhizobium* interaction, the bacteria released from the infection thread are surrounded by a plant-derived membrane, forming a symbiosome [68]. Laser capture microdissection of *Medicago truncatula* root nodules showed that a specific zone of the nodule contained these symbiosomes in which N-fixation takes place [69]. The nature of these structures in the non-legume–diazotrophic endophyte symbiosis is yet to be understood, but it is possible that they allow nitrogen fixation in an aerobic environment by creating a barrier to oxygen diffusion [56]. Future studies will explore the specificity of the nitrosomes in the poplar–diazotrophic endophyte interaction.

The nitrosomes *in planta* and the high expression of aggregated cells *in vitro* both point to a structural component element to the needed conditions for nitrogen fixation. It seems likely that QS would be involved in inducing N-fixation but it is unclear whether QS leads to structural changes, such as EPS production, or is triggered by it. It is clear that, similar to other N-fixation systems and structures found in nature, N-fixation by WPB occurs only in a subset of the population, hinting at differentiated and complimentary metabolic roles. Here, this strain was studied in isolation, but interspecies interactions are likely to take place. These findings show a dynamic community of cells is present and likely needed for successful N-fixation by WPB both *in vitro* and in association with its host plant.

## Supplementary Material

Rev_Supplementary_Files_wrad012

Supplementary_Video_wrad012

## Data Availability

Materials will be freely available to any researcher for noncommercial purposes. The accession number for the WPB genomic sequence is GCA_9001022765.1. The DOI for the metabolomics and proteomics data is DOI 10.17605/OSF.IO/DHUZN—which is hosted on OSF @ https://osf.io/dhuzn.
