# Supplementary material for: Dynamic nitrogen fixation in an aerobic endophyte of Populus
Source: ISME J. 2024 Jan 10;18(1):wrad012. doi: 10.1093/ismejo/wrad012 (PMC10833079; doi:10.1093/ismejo/wrad012)
Supplement: Rev_Supplementary_Files_wrad012 [file rev_supplementary_files_wrad012.pdf]

Supplementary Figures

Supplementary Figure 1. Re-formation of spatial patterning on nitrogen limited plates by pools of 7 μl from an OD_{600} 0.4 cell suspension. Cell suspensions from regions expressing *nifH* indicated by +, suspensions from regions not observed to be expressing *nifH* indicated by -. **A)** Spatial patterning after one week. **B)** Spatial patterning is persistent after 2 weeks.

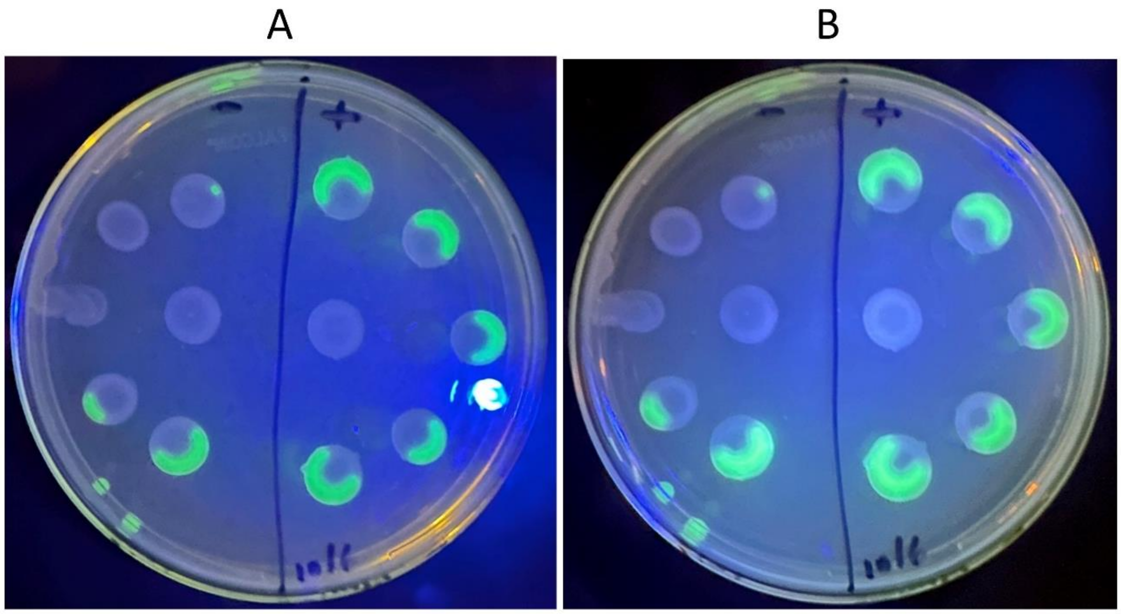

Supplementary Figure 2: Fluorescence Activated Cell Sorting (FACS) measured the relative abundance of GFP from WPB Pnif-GFP to be 11.5% of the total population (50,000 recorded cells)

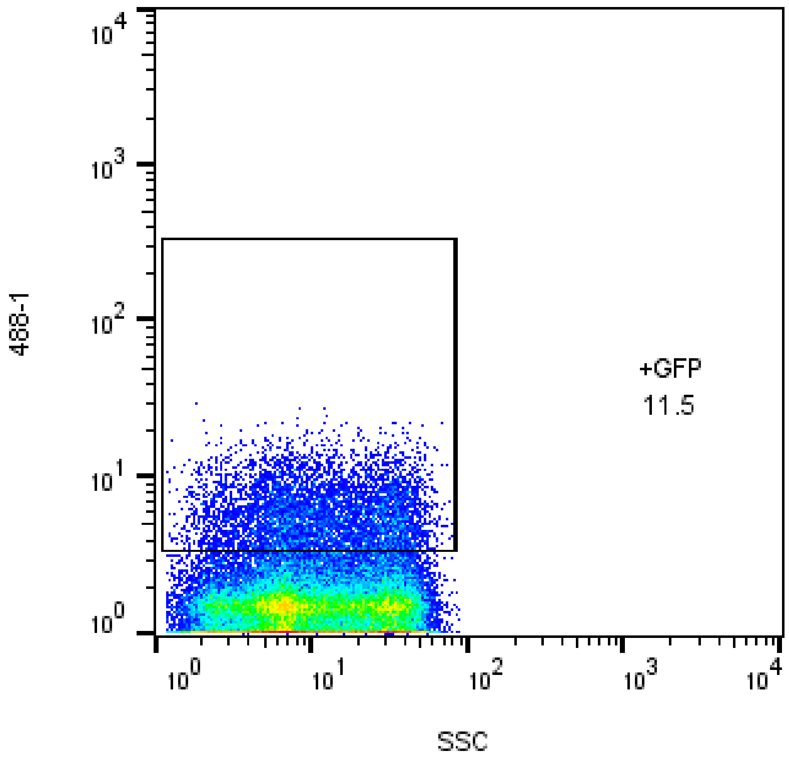

Supplementary Figure 3: A) *P. trichocarpa* cuttings were transferred to the RhizoChip platform, a synthetic soil habitat constructed from transparent polydimethylsiloxane that is compatible with optical imaging, where they were co-cultured with WPB cells. A representative image of a *P. trichocarpa* plant after growing in the RhizoChip for B) 17 days and C) 31 days.

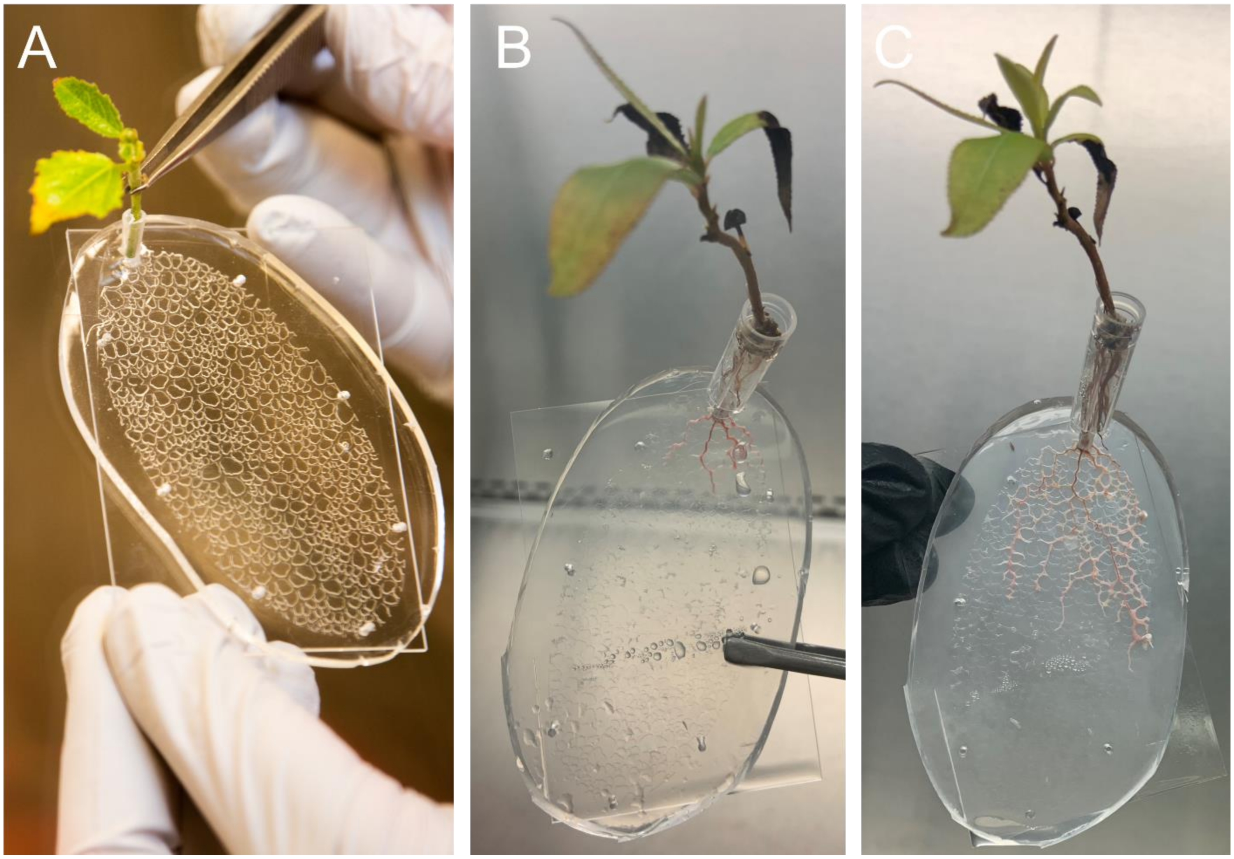

Supplementary Figure 4. Post-NanoSIMS scanning electron micrograph of replicate 15N-3 (top) and individual hue saturation intensity (HSI) NanoSIMS images representing individual atom% 15N. Scale bar represents 35 μm.

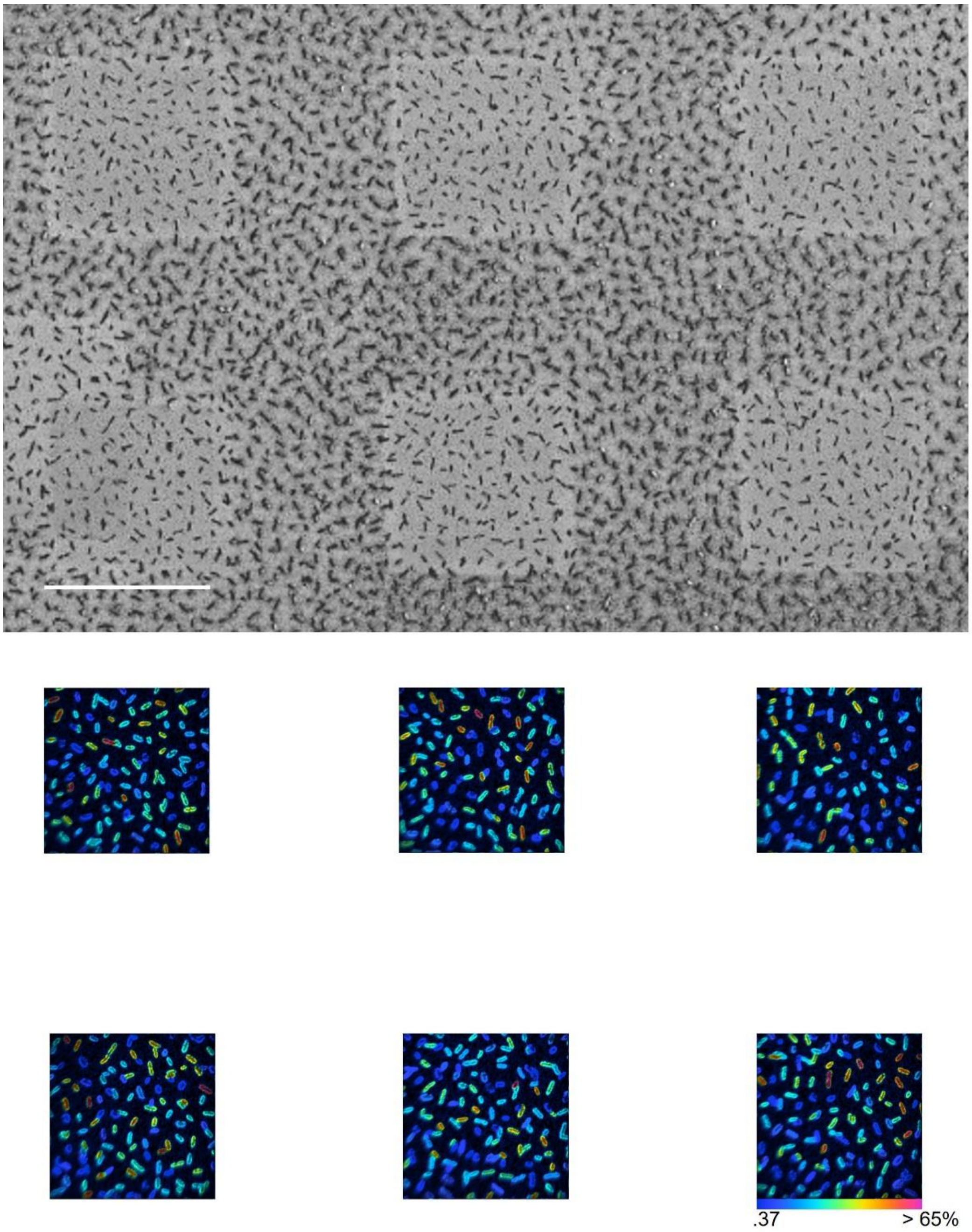

Supplementary Video 1: 3D images of *nif*H expressing WPB cells (green dots) inside of *P. trichocarpa* root epidermal cells observed using a lattice lightsheet microscope. [Still photo shown here]

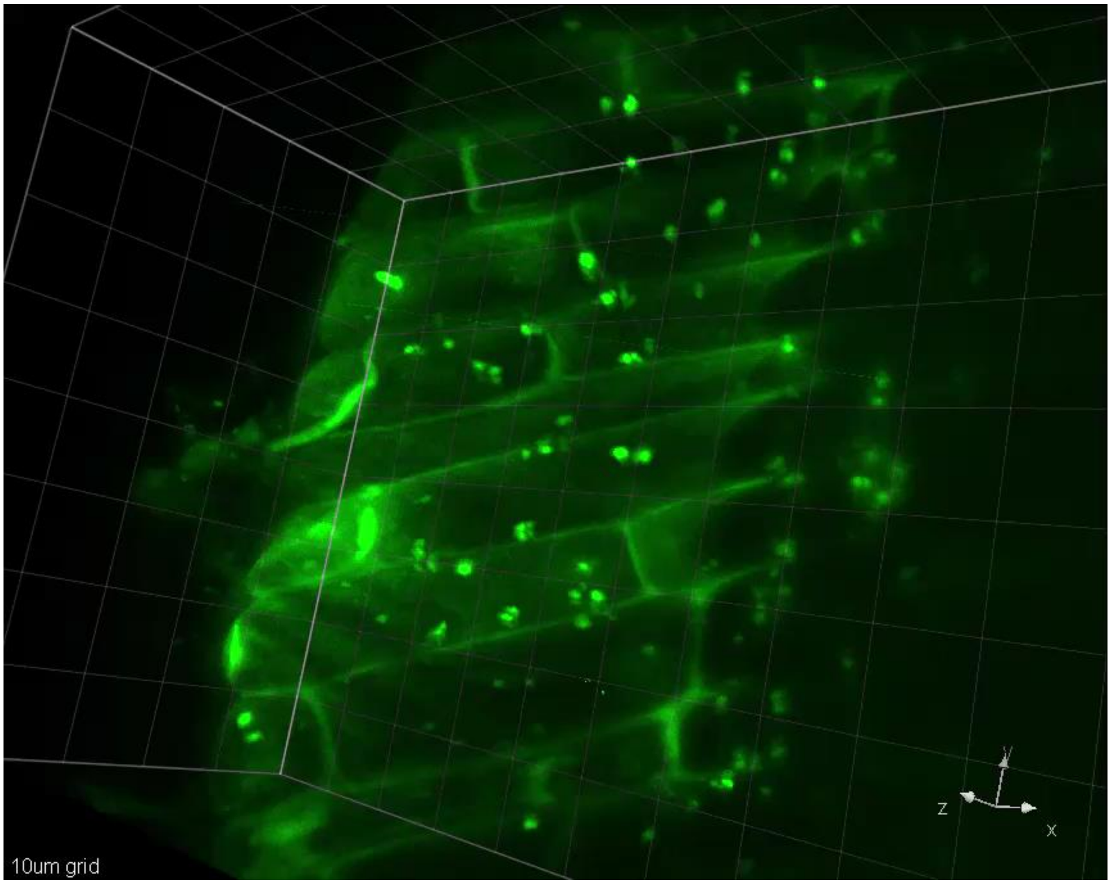

Supplementary Tables

Supplementary Table 1. Table of NanoSIMS ^{15}N estimates of individual bacteria for the *in vitro* $^{15}N_2$ fixation experiment

Sample	valine	ethanolamine	leucine	isoleucine	serine	threonine	pyroglutamic acid	GABA	glutamic acid	phenylalanine	putrescine	O-phosphoethanolamine	ornithine	cadavarine	lysine	tyrosine
T3_01_crl_M	0.0367	0.015	0.0313	0.0091	0.0085	0.015	0.0076	0.0136	0.0043	0.0096	0.01	-0.0038	0.0474	0.0116	0.01	0.0083
T3_02_crl_M	0.0453	0.0077	0.0619	0.0195	0.0066	0.0017	0.0221	0.0137	0.0089	0.0225	-0.0013	0.0408	0.0117	0.0114	0.0289	0.0135
T3_03_crl_M	0.0326	-0.0114	0.0338	0.0113	0.0053	0.0083	0.0142	0.0187	0.0066	0.0127	0.0052	0.0293	0.0012	0.0057	0.0424	0.0059
T3_04_crl_M	0.0357	-0.01	0.0569	0.0152	0.0069	0.0094	0.0114	0.0257	0.0105	0.0096	0.0055	0.0026	0.0228	0.0097	0.0247	0.0088
T3_05_crl_M	0.0471	0.0087	0.0804	0.0227	0.0079	0.0074	0.018	0.0229	0.0087	0.0111	0.0065	0.035	0.0202	0.0114	0.0188	0.0088
T3_06_crl_M	0.0322	0.001	0.0468	0.0328	0.0068	0.015	0.0156	0.0399	0.0175	0.0177	0.001	0.0191	0.0105	0.0079	0.0273	0.0056
T3_07_15N_M	0.0928	0.0283	0.128	0.0728	0.0511	0.0168	0.0757	0.0658	0.0657	0.0495	0.0908	0.0473	0.0632	0.0405	0.0797	0.0522
T3_08_15N_M	0.0996	0.0089	0.1242	0.0591	0.0564	0.0237	0.0729	0.0605	0.0625	0.0651	0.0661	0.0374	0.0609	0.0371	0.0714	0.058
T3_09_15N_M	0.0974	0.0014	0.0942	0.0474	0.0563	0.0333	0.0753	0.0724	0.0816	0.057	0.1139	0.0827	0.0294	0.0416	0.0808	0.0574
T3_10_15N_M	0.0771	0.0067	0.1071	0.0487	0.0466	0.0151	0.0652	0.0737	0.0534	0.0465	0.0916	0.0571	0.0208	0.0331	0.0472	0.0452
T3_11_15N_M	0.1102	-0.0006	0.1082	0.0727	0.0664	0.0434	0.0862	0.0803	0.0749	0.0715	0.0718	0.0628	0.0617	0.0464	0.1168	0.0824
T3_12_15N_M	0.1176	-0.0008	0.1389	0.0773	0.0739	0.0562	0.0956	0.093	0.0821	0.0933	0.0683	0.0595	0.0765	0.0467	0.0821	0.082
T9_13_crl_M	0.0253	0.0203	0.1556	0.091	0.0098	0.011	0.0278	-0.0001	0.0082	0.0679	0.004	0.0162	0.0618	0.0045	-0.2527	0.0314
T9_14_crl_M	0.0314	-0.0074	0.0773	0.0575	0.0087	0.0015	0.0377	-0.0054	0.0164	0.0499	0.0078	0.0066	0.051	0.0077	0.0045	0.0143
T9_15_crl_M	0.0423	-0.0098	0.0211	0.0225	0.0038	0.0252	0.0153	0.0086	-0.0049	0.0379	0.0008	0.0111	-0.0321	0.0065	-0.0159	0.0176
T9_16_crl_M	0.0271	-0.0063	0.0473	0.0508	0.0077	0.0172	0.022	-0.0087	0.006	0.0286	0.0106	0.0366	0.0488	0.0047	0.0135	0.0088
T9_17_crl_M	0.0272	-0.0001	0.071	0.0188	0.0058	-0.0005	0.0354	0.0126	-0.0036	0.0667	0.0014	-0.0021	-0.0325	0.0058	-0.0023	0.0188
T9_18_crl_M	0.0448	0.0137	0.0862	0.0615	0.0065	-0.0098	0.011	0.0162	0.0098	0.0691	0.0101	0.0197	0.0369	0.0083	0.1093	0
T9_19_15N_M	0.1174	0.0037	0.1301	0.1498	0.0844	0.0833	0.1036	0.149	0.0757	0.1037	0.1961		0.144	0.0608	0.0982	0.0675
T9_20_15N_M	0.0945	0.0065	0.1146	0.0931	0.064	0.0501	0.0788	0.0683	0.0602	0.1249	0.0962	0.0749	0.0747	0.0647	0.0805	0.084
T9_21_15N_M	0.1039	0.0033	0.1634	0.0936	0.0639	0.052	0.0781	0.0737	0.0539	0.1187	0.083	0.0659	0.0576	0.0561	-0.1608	0.0622
T9_22_15N_M	0.118	0.0008	0.1976	0.0787	0.0622	0.03	0.0904	0.0766	0.0604	0.1035	0.0897	0.0733	0.0706	0.0524	0.0545	0.0676
T9_23_15N_M	0.1066	0.0014	0.1144	0.081	0.0734	0.0365	0.0919	0.0955	0.0749	0.1299	0.1105	0.084	0.0685	0.0637	0.0942	0.0738
T9_24_15N_M	0.1147	0.0051	0.1587	0.1008	0.0842	0.0884	0.1022	0.0907	0.0868	0.1171	0.1256	0.0716	0.0913	0.0852	0.1087	0.0982
